# Frequency-Domain Fusing Convolutional Neural Network: A Unified Architecture Improving Effect of Domain Adaptation for Fault Diagnosis

**DOI:** 10.3390/s21020450

**Published:** 2021-01-10

**Authors:** Xudong Li, Jianhua Zheng, Mingtao Li, Wenzhen Ma, Yang Hu

**Affiliations:** 1 National Space Science Center.CAS, University of Chinese Academy of Sciences, Beijing 100190, China; lixudong16@mails.ucas.edu.cn (X.L.); zhengjianhua@nssc.ac.cn (J.Z.); limingtao@nssc.ac.cn (M.L.); mawenzhen@nssc.ac.cn (W.M.); 2 Science and Technology on Complex Aviation System Simulation Laboratory, Beijing 100076, China

**Keywords:** fault diagnosis, domain adaptation, frequency domain, convolutional neural network, dilated convolution

## Abstract

In recent years, transfer learning has been widely applied in fault diagnosis for solving the problem of inconsistent distribution of the original training dataset and the online-collecting testing dataset. In particular, the domain adaptation method can solve the problem of the unlabeled testing dataset in transfer learning. Moreover, Convolutional Neural Network (CNN) is the most widely used network among existing domain adaptation approaches due to its powerful feature extraction capability. However, network designing is too empirical, and there is no network designing principle from the frequency domain. In this paper, we propose a unified convolutional neural network architecture from a frequency domain perspective for a domain adaptation named Frequency-domain Fusing Convolutional Neural Network (FFCNN). The method of FFCNN contains two parts, frequency-domain fusing layer and feature extractor. The frequency-domain fusing layer uses convolution operations to filter signals at different frequency bands and combines them into new input signals. These signals are input to the feature extractor to extract features and make domain adaptation. We apply FFCNN for three domain adaptation methods, and the diagnosis accuracy is improved compared to the typical CNN.

## 1. Introduction

Modern machinery and equipment are widely used in industrial production, and their structures are sophisticated and complex. They are usually operated in a high-intensity working environment. Among them, rotating machinery plays an essential role in modern mechanical equipment, and is fragile and vulnerable to damage, significantly affecting the entire system’s stability. Therefore, fault diagnosis of rotating machinery is vital in the modern industry. To get better diagnosis results, it is critical to extract significant features. Traditional data-driven fault diagnosis methods extract features artificially from raw signals, namely handcraft features [1,2,3]. These handcraft features can be generated from time domain, frequency domain, time-frequency domain or other signal processing methods, and are classified by pattern recognition algorithms, such as Support Vector Machine (SVM) [4,5], K-nearest Neighbors (k-NN) [6], Decision Tree (DT) [7,8] and so on. However, handcraft features require a lot of experience and professional knowledge, and different problems may require different feature extraction methods. Besides, feature selection among variously alternative features is also tricky and time-consuming.

In recent years, deep learning has been applied in fault diagnosis [9,10,11], which has a powerful ability to learn features from large amounts of data compared with traditional machine learning [12]. It can automatically mine useful features from signals and regularization terms can be added for feature selection. Besides, deep learning can achieve end-to-end learning that combines feature extraction and classification. The feature extraction and classifier of traditional methods are uncoupled and independent from each other. But feature extractor and classifier of deep learning are trained jointly, and the extracted features are specific to certain diagnostic tasks [13].

While deep learning has achieved good performance in fault diagnosis, two problems need to be solved: (a) Exiting deep learning models require a lot of labeled data. However, sensors of industrial devices will produce a lot of unlabeled data in a short time, and labeling data is very time-consuming and labor-intensive [14]. (b) Operating conditions of actual industrial equipment are often changing, which results in different distributions of collected datasets [15]. a model trained on one specific dataset will have poor generalization ability on another dataset with a different distribution.

To solve the above problems, transfer learning, a branch of machine learning, has been employed in fault diagnosis [16]. In transfer learning, the domain has a lot of labeled data and knowledge is called the *source domain*, and the *target domain* is the object that we want to transfer knowledge to [17,18]. Based on whether the source domain dataset has labels, transfer learning is divided into three categories: supervised transfer learning, semi-supervised transfer learning and unsupervised transfer learning [17]. In this paper, we focus on unsupervised transfer learning. a widely used method to solve unsupervised transfer learning is *domain adaptation*, which is to learn common feature expressions between two domains to achieve feature adaptation [19,20]. Domain adaptation has been proven effective in fault diagnosis and has become one of the research hot spots in fault diagnosis [16]. However, exciting domain adaptation methods for fault diagnosis extract features on a single scale, and do not consider network design from the perspective of frequency-domain. In this paper, amplitude-frequency characteristics (AFC) curve is utilized to describe the frequency domain characteristics of convolution kernels for the first time. Inspired by the discovery that convolution kernels of different scales filter signals of different frequency bands, we propose a unified CNN architecture to improve the effect of domain adaptation for fault diagnosis, named Frequency-domain Fusing CNN (FFCNN). Since a large kernel will increase the number of the networks’ parameters, we use dilated convolution [21,22,23] to expand the receptive field of convolution kernel without increasing the number of parameters. FFCNN concatenates several convolution kernels with different dilation rates in the first layer, which will extract features at different scales of the original signals. Then these features are fused for domain adaptation.

While some papers have proposed similar network architectures of multi-scale convolution [24,25,26,27], our approach differs from theirs in the following respects: (a) Most existing papers focus on general classification problems, but we have verified the effectiveness of multi-scale structure in domain adaptation; (b) Most methods do not clarify the physical meaning of multi-scale convolution, but our method is driven by the frequency-domain characteristics of convolution kernels, which has a clear physical meaning. Compared with the previous domain adaptation methods for fault diagnosis, our proposed method is unified and suitable for different domain adaptation losses. In consequence, the contributions of this paper are summarized as follows:

1. We design the network architecture for fault diagnosis from the perspective of frequency-domain characteristics of convolution kernels. The motivation for network design has a clear physical meaning.
2. For the first time, we use the amplitude-frequency characteristic curve to describe the frequency domain characteristic of the convolution kernels. This provides a new idea for analyzing the physical meaning of the convolution kernels.
3. the proposed FFCNN is suitable for various domain adaptation loss functions, and can significantly improve the performance of domain adaptation for fault diagnosis without increasing the complexity of the networks.
4. Dilated convolution is used in domain adaptation and fault diagnosis. Dilated convolution can improve the receptive field without increasing the number of parameters.

The rest of this paper is organized as follows. In Section 2, related work about deep learning methods and domain adaptation methods are introduced. Some background knowledge will be introduced, including domain adaptation, CNN, and dilated convolution in Section 3. Section 4 will give the motivation of our proposed method. Section 5 will detail the proposed MSCNN and the training process. Section 6 will study two cases and provide in-depth analysis from different perspectives. Some usage suggestions, existing problems and future research contents are given in Section 7. Finally, the conclusions are drawn in Section 8. The symbols used in this paper are listed in Abbreviations.

## 2. Related Work

Deep learning for fault diagnosis. a variety of deep learning methods have been successfully applied in fault diagnosis in recent years. Jia et al. [28] proposes a Local Connection Network (LCN) constructed by normalized sparse Autoencoder (NSAE), named NSAE-LCN. This method overcomes two shortcomings of traditional methods: (a) They may learn similar features in feature extraction. (b) the learned features have shift variant properties, which leads to the misclassification of fault types. Yu et al. [29] proposed a component selective Stacked Denoising Autoencoders (SDAE) to extract effective fault features from vibration signals. Then correlation learning is used to fine-tune the SDAE to construct component classifiers. Finally, a selective ensemble is finished based on these SDAEs for gearbox fault diagnosis. Except for autoencoder, CNN is also a widely used deep learning method. Jing et al. [30] developed a 1-D CNN to extract features directly from frequency data of vibration signals. The results showed that the proposed CNN method can extract more effective features than the manually-extracting method. Huang et al. [27] developed an improved CNN that uses a new layer before convolutional layer to construct new signals of more distinguishable information. The new signals are obtained by concatenating the signals convolved by kernels of different lengths. Generative adversarial network (GAN) and Capsule Network (CN) are the latest research results of deep learning. Han et al. [31] used adversarial learning as a regularization in CNN. The adversarial learning framework can make the feature representation robust, boost the generalization ability of the trained model, and avoid overfitting even with a small size of labeled data. Chen et al. [32] proposed a novel method called deep capsule network with stochastic delta rule (DCN-SDR). The effective features are extracted from raw temporal signals, and the capsule layers reserve the multi-dimensional features to improve the representation capacity of the model.

Domain adaptation for fault diagnosis. Domain adaptation method can use the unlabeled data for transfer learning. In the work of Li et al. [33], the multi-kernel maximum mean discrepancies (MMD) are minimized to adapt the learned features in multiple layers between two domains. This method can learn domain-invariant features and significantly improve the performance of cross-domain testing. Han et al. [34] proposed an intelligent domain adaptation framework for fault diagnosis, deep transfer network (DTN). DTN extends the marginal distribution adaptation to joint distribution adaptation, guaranteeing a more accurate distribution matching. Wang et al. [35] applies adversarial learning to domain adaptation, and proposes Domain-Adversarial Neural Networks (DANN). In addition, a unified experimental protocol for a fair comparison between domain adaptation methods for fault diagnosis is offered. Guo et al. [36] proposes an intelligent method named deep convolutional transfer learning network (DCTLN) consists of condition recognition and domain adaptation. The condition recognition module is a 1-D CNN to learn features and recognize machines’ health conditions. The domain adaptation module maximizes domain recognition errors and minimizes probability distribution distance to help 1-D CNN learning domain invariant features. Li et al. [37] proposed a weakly supervised transfer learning method with domain adversarial training. This method aims to improve the diagnostic performance on the target domain by knowledge transferation from multiple different but related source domain.

## 3. Background

### 3.1. Transfer Learning and Domain Adaptation

We consider a deep learning classification task T where $X=\{x_{1},x_{2},\cdots ,x_{n}\}$ is the dataset sampled form input space X and $Y=\{y_{1},y_{2},\cdots ,y_{n}\}$ is the labels of dataset from label space Y. Above elements form a specific *domain* D. We need to learn a feature extractor g(·):X→Z and a classifier h(·):Z→Y, where *Z* is the learned features representation. Given two domains with different distributions named *source domain* $D^{S}$ and *target domain* $D^{T}$, *transfer learning* is to improve the performance of target domain using the knowledge of source domain, where $X^{S}\neq X^{T}$ or $Y^{S}\neq Y^{T}$.

From the perspective of input spaces and label spaces, transfer learning can be divided into the following two types:

- Homogeneous transfer learning. The input spaces of the source domain and target domain are similar and the label spaces are the same, expressed as $X^{S}\cap X^{T}\neq \emptyset$ and $Y^{S}=Y^{T}$.
- Heterogeneous transfer learning. Both the input spaces and the label spaces may be different, expressed as $X^{S}\cap X^{T}=\emptyset$ or $Y^{S}\neq Y^{T}$.

Besides, according to whether the target domain contains labels, transfer learning can also be divided into following three types:

- Supervised transfer learning. All data in the target domain have labels.
- Semi-supervised transfer learning. Only part of the data in the target domain have labels.
- Unsupervised transfer learning. All data in the target domain have no labels.

Most of the research in recent years has focused on unsupervised homogeneous transfer learning [38], which is also the direction of our work. Domain adaptation is a common method to solve unsupervised homogeneous transfer learning. Given source domain $D^{S}$ and target domain $D^{T}$, a labeled source dataset $X^{S}$ is sampled i.i.d from $D^{S}$, and an unlabeled target dataset $X^{T}$ is sampled i.i.d form $D^{T}$. a domain adaptation problem aims to train a common feature extractor g(·):X→Z over $X^{S}$ and $X^{T}$, and a classifier h(·):Z→Y learned from $X^{S}$ with a low target risk [39]:

(1) $$err_{D^{T}}\left(h\right)={\mathrm{Pr}}_{\left(x,y\right)∼D_{T}}\left(h(g(x))\neq y\right)$$

To adapt the feature space of source domain and target domain, a specific criterion $d(Z^{S},Z^{T})$ is chosen for measuring the discrepancy between $Z^{S}$ and $Z^{T}$. which is regarded as a loss function.

### 3.2. Convolutional Neural Network

In this paper, a one-dimensional convolutional neural network is built to extract features and classify fault types. a typical CNN consists of convolution layers, pooling layers and a fully-connected layer. Let $x_{i}^{l-1}=\left\{x_{i}^{l-1,S},x_{i}^{l-1,T}\right\}\in R^{N\times M}$ is the output of ${\left(l-1\right)}^{th}$ layer containing source domain data and target domain data, *N* is the number of channels, *M* is the dimensional of feature maps. The kernel of $l^{th}$ convoluntion layers is $k^{l}\in R^{C\times N\times H}$, bias is $b^{l}\in R^{C}$, *C* is the number of channels in the output feature maps, *H* is kernel size. So the output of $l^{th}$ layer is obtained as follows [13]:

(2) $$\begin{array}{cc} x_{i,(\mathrm{oonv})}^{l} & =\sigma \left(x_{i}^{l-1}*k^{l}+b^{l}\right)\in R^{N^{\prime }\times M^{\prime }}\\ N^{\prime } & =C\\ M^{\prime } & =\left\lfloor \frac{M-H+p}{s}+1\right\rfloor \end{array}$$

where σ(·) is activation function, * is convolution operation, *s* is the stride step, and *p* is padding size to keep the input and output dimensions consistent. After convolution layer, a down-sampling layer is connected to reduce the number of parameters and avoid overfitting [13]:

(3) $$\begin{array}{cc} x_{i,(pool)}^{l} & =\mathrm{pool}\left(x_{i}^{l}\right)\in R^{N^{\prime \prime }\times M^{\prime }}\\ N^{\prime \prime } & =C\\ M^{\prime \prime } & =\left\lfloor \frac{M^{\prime }-L}{s}+1\right\rfloor \end{array}$$

where *s* is the pooling step, and *L* is pooling size. Repeat convolution layer and pooling layer several times to deepen the network. Then the feature maps are flattened into one-dimension to connect a fully-connected layer. Finally, the softmax layer outputs the predicted classification probability:

(4) $$\begin{array}{cc} x_{i}^{l} & =\;\mathrm{flatten}\;\left(x_{i}^{l-1}\right)\\ {\tilde{y}}_{i} & =\mathrm{soft}\max\left(\sigma \left(w_{1}x_{i}^{l}+b_{1}\right)\right) \end{array}$$

The classification loss used to measure the discrepancy between predictions and labels can be expressed by cross-entropy:

(5) $$\ell_{\mathrm{clf}}\left(y,\tilde{y}\right)=\frac{1}{m}\sum_{i=1}^{m}\left(-y_{i}\cdot \log\left({\tilde{y}}_{i}^{⊤}\right)-\left(1-y_{i}\right)\cdot \log\left(1-{\tilde{y}}_{i}^{⊤}\right)\right)$$

where $y_{i}$ is the real label of $i^{th}$ sample. The objective of the classification task is to optimize the loss function to reduce the classification risk.

### 3.3. Dilated Convolution

**Figure 1:**
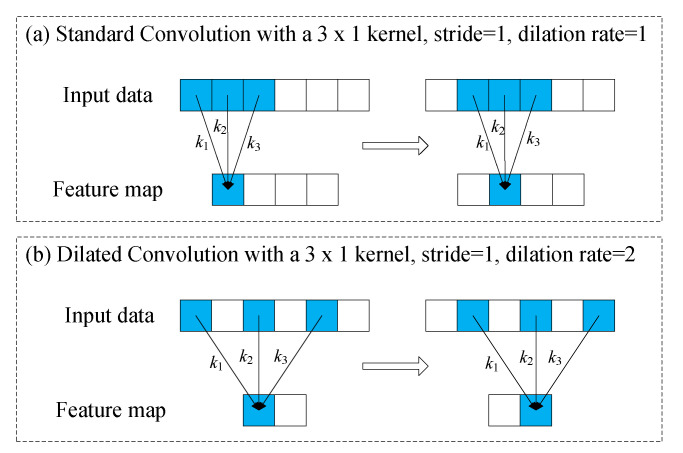
Comparison of standard convolution and dilated convolution.

To explain dilated convolution, we compare it with a standard convolution as shown in Figure 1. We assume that the input data $x=[x_{1},x_{2},x_{3},x_{4},x_{5},x_{6}]$ is six-dimensions, kernel is $k=[k_{1},k_{2},k_{3}]$, stride is 1. According to Equation (1), the output is $x^{\prime }=\left[x_{1}^{\prime },x_{2}^{\prime },x_{3}^{\prime }\right]$ in Figure 1a, where

(6) $$x_{j}^{\prime }=x_{j}k_{1}+x_{j+1}k_{2}+x_{j+2}k_{3}+b$$

In the standard convolution, the adjacent elements of the input data are multiplied and added to the kernel, and the operation is repeated by sliding *s* strides to the end of input data. Dimension of output is $\left\lfloor \frac{6-3}{1}+1\right\rfloor =4$.

In dilated convolution, we denote *r* the dilation rate. Unlike standard convolution, the elements multiplied and added with the kernel are separated by r−1 elements in dilated convolution. In Figure 1b, dilation rate is 2, and the output becomes $x^{\prime }=\left[x_{1}^{\prime },x_{2}^{\prime }\right]$ [21], where

(7) $$x_{j}^{\prime }=x_{j}k_{1}+x_{j+r}k_{2}+x_{j+2r}k_{3}+b.$$

Dilated convolution is equivalent to expanding the kernel size, that is, expanding the receptive field, and the equivalent kernel size is [40]:

(8) $$H_{dilated}=H+\left(H-1\right)\left(r-1\right)$$

So the dimension of output $M^{\prime }$ becomes:

(9) $$M^{\prime }=\left\lfloor \frac{M-H\times r+r+p-1}{s}+1\right\rfloor$$

The standard convolution is the dilated convolution of r=1.

## 4. Motivation

**Figure 2:**
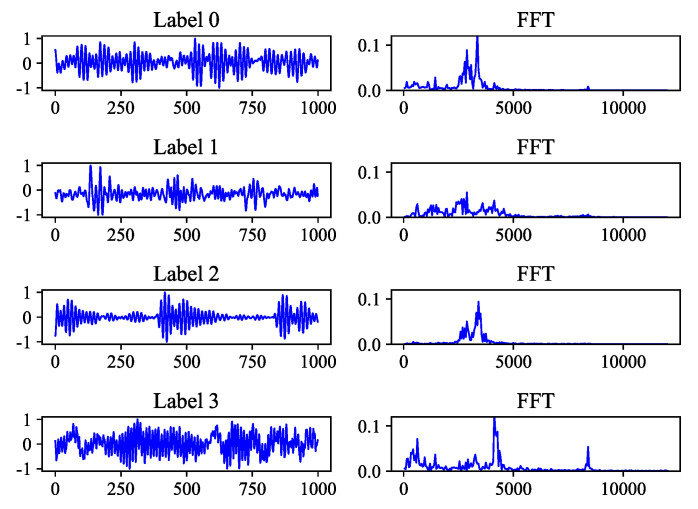
Vibration signal samples of different fault type and their FFT spectrograms.

The vibration signal is time domain signal, and most deep learning methods are designed from the perspective of time domain. But vibration signal can be composed of a series of sine wave signals with different frequencies, phases, and amplitudes, which are the frequency domain representations of the vibration signal. The vibration modes of different fault types are different, and the FFT spectrograms are also different, as shown in Figure 2. Signals of different fault type have different dominant frequency bands, which means that useful information is contained in different frequency bands. Traditional methods usually use some signal processing techniques to extract features in the time domain and frequency domain. The commonly used CNN can automatically extract features from the original signals and learn related fault modes based on the labeled data. But what exactly does the learned convolution kernel mean? Here we can regard the first layer of convolution kernels as the preprocessing of the original signals. To observe the frequency domain characteristics of the convolution kernels, we can draw the amplitude-frequency characteristics (AFC) curve of kernels. Next, the principle of AFC will be explained.

Let the input signal is x, the output signal after a convolutional kernel is $\tilde{x}$, and the convolution operation can be seen as a function G(·). To get the AFC curve of G(·), we take a series of sinusoidal signals $X=\{x_{1},x_{2},\cdots ,x_{i},\cdots ,x_{m}\}$ with different frequencies $\left\{f_{1},f_{2},\cdots ,f_{i},\cdots ,f_{m}\right\}$. For each signal, the length is $n_{t}$:

(10) $$\begin{array}{cc} \; & x_{i}=\left[x_{i}^{1},x_{i}^{2},\ldots ,x_{i}^{t},\ldots ,x_{i}^{n_{t}}\right]\\ \; & x_{i}^{t}=\sin\left(2\pi \cdot f_{i}\right) \end{array}$$

Then a series of corresponding outputs $\tilde{X}=\left\{{\tilde{x}}_{1},{\tilde{x}}_{2},\cdots ,{\tilde{x}}_{i},\cdots ,{\tilde{x}}_{m}\right\}$ will be obtained. The amplitude ratio of the output signal to the input signal is calculated, and the logarithm of 20 times is taken:

(11) $$A\left(f_{i}\right)=20\mathrm{lg}\left(\frac{\left|G\left(x_{i}\right)\right|}{\left|x_{i}\right|}\right)$$

where $\left|G(x_{i})\right|$ is amplitude of output signal, $|x_{i}|$ is amplitude of the input signal. So we will get a set of $\left\{f_{i}\to A\left(f_{i}\right)|i=1,2,\cdots ,m\right\}$. With $f_{i}$ from low to high as the horizontal axis and $A(f_{i})$ as the vertical axis, we can get the AFC curve. AFC curve shows the ability of a convolution kernel to suppress signals in various frequency bands. In general, the signal amplitude that passes through the filter will decrease and $A(f_{i})$ will be negative. If the value $A(f_{i})$ is very small, the filter will suppress the signal $x_{i}$ with frequency $f_{i}$. In contrast, the filter does not suppress the signal $x_{i}$.

**Figure 3:**
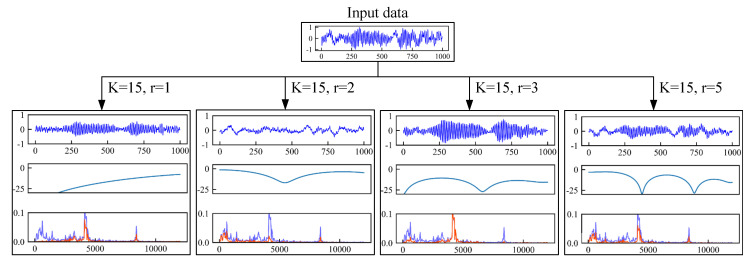
Several typical amplitude-frequency characteristic curves and the signals after convolution without activation function. K is the kernel size, and *r* is the dilation rate. In the four parallel subgraphs below, the first row is the output of signal after convolution, the second row is the amplitude-frequency characteristics (AFC) curve, and the third row is the FFT spectrogram. In FFT spectrogram, the blue line represents the original signal, the red line represents the output signal.

To explore the meaning of the convolution kernel from a frequency domain perspective, we trained four CNN with different kernel sizes (kernel size is 15, dilation rates are 1, 2, 3, and 5). The output of signal after the first convolution layer, AFC curve of one of the convolution kernels and FFT spectrogram of output are drawn in Figure 3. As we can see that the convolution kernels can be regarded as a series of filters, which can filter out signals of different frequency bands. Observing these AFC curves, we can get the following points:

- the convolution kernels can be regarded as a series of filters, which can suppress signals in some single frequency bands.
- Different dilation rates have different AFC curves. Convolution kernels with a dilation rate r>1 have multiple suppression bands. And kernels with higher dilation rates have more suppression bands.

The above findings motivate us to design the network architecture from the perspective of the frequency domain. We change the first layer of CNN to a multi-scale convolution kernel fusion method. The input signal is preprocessed in multiple frequency bands before entering the next stage of feature extraction. Compared with single-scale CNN, the improved CNN can extract richer frequency domain information to improve CNN’s feature extraction ability.

## 5. Proposed Method

### 5.1. Frequency-Domain Fusing CNN

**Figure 4:**
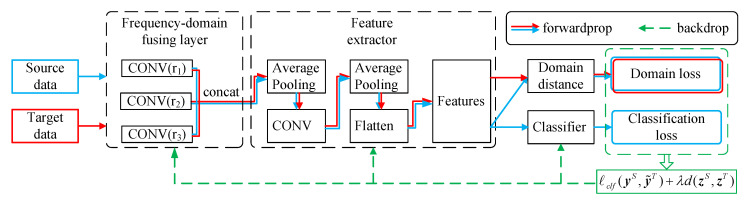
Architecture of proposed FFCNN.

The architecture of the proposed FFCNN is shown in Figure 4. Note that the depth of the network should match the size of dataset. a small network will cause underfitting, while a large network will easily cause overfitting and increase training time. According to the size of dataset used in this paper and some hyper-parameter debugging experiments, we used a CNN including two convolution layers and two fully-connected layers. The details of FFCNN used in this paper are shown in Table 1. For dilation rates, although a large dilation rate will expand receptive field, it is not the bigger the better. According to the debugging experiments, we have selected two sets of dilation rates with appropriate sizes, r=1,2,3 and r=1,3,5, to evaluate the effect of different dilate rates. Section 6.3 and Section 6.4 will discuss the effect of different dilation rates.

For FFL, there are three convolutional branches with different dilation rates in the first convolution layer. They can preprocess signals on multiple scales and produce feature maps with the same number of channels and dimensions. Then the three feature maps are connected in the channels axis and followed by a pooling layer. For example, there are three convolution layers with dilation rate r=1,2,3 that produce three feature maps with *C* channels and *N* dimensions, and the three feature maps are connected to a feature map with the shape of 3C×N. Next, the feature map is followed by standard convolution layers and pooling layers, a feature extractor of the second stage. Then the final convolution layer’s feature map is flattened and followed by fully-connected layers. Finally, the classification loss and domain loss are obtained.

For domain adaptation, the source data $X^{S}$ and target data $X^{T}$ are trained jointly. Source data and target data are mapped to source features $Z^{S}$ and target features $Z^{T}$ by the feature extractor. The discrepancy measured by $d(Z^{S},Z^{T})$ between $Z^{S}$ and $Z^{T}$ is calculated as a domain adaptation loss, $Z^{S}$ is classified by softmax layer and classification loss is obtained. Domain loss and classification loss together are optimized as a total loss. Back propagation (BP) algorithm is used to upgrade each layer’s parameters until the loss converges or reaches the maximum number of iteration.

### 5.2. Learning Process

Let $X^{S}={\left\{x_{i}^{S},y_{i}^{S}\right\}}_{i=1}^{n_{s}}$ be the labeled source domain dataset, $X^{T}={\left\{x_{i}^{T}\right\}}_{i=1}^{n_{T}}$ be the unlabeled target domain dataset. The parameters set of the three branches in the first dilated convolution layer is $\theta_{r_{j}}^{conv1}=\left\{k_{r_{j}}^{conv1},b_{r_{j}}^{conv1}|j=1,2,3\right\}$, the output feature maps after dilated convolution and maxpooling are:

(12) $$x_{i,r_{j}}^{conv1}=\mathrm{pool}\left(\sigma \left(x_{i}*k_{r_{j}}^{conv1}+b_{r_{j}}^{conu1}\right)\right)\in R^{C_{1}\times M_{1}}$$

where $x_{i}=\left\{x_{i}^{S},x_{i}^{T}\right\}$ containing source and target domain data. They are connected into one feature map $x_{i}^{conv1}=\mathrm{concat}\left(\left\{x_{i,r_{j}}^{conv_{1}}|j=1,2,3\right\}\right)\in R^{3C_{1}\times M_{1}}$ by channels. The feature map is followed by the second convolution layer and maxpooling layer with parameters $\theta^{conv2}=\left\{k^{conv2},b^{conv2}\right\}$ and flatten:

(13) $$\begin{array}{c} x_{i}^{conv2}=\mathrm{pool}\left(\sigma \left(x_{i}^{conv1}*k^{conv2}+b^{conv2}\right)\right)\in R^{C_{2}\times M_{2}}\\ x_{i}^{flatten}=\mathrm{flatten}\;\left(x_{i}^{conv2}\right) \end{array}$$

Next a fully-connected layer with parameters $\theta^{fc}=\left\{w_{1},b_{1}\right\}$ and $\theta^{clf}=\left\{w_{2},b_{2}\right\}$ is followed to extract feature representations and classify them:

(14) $$\begin{array}{cc} z_{i} & =\sigma \left(w_{1}x_{i}^{flatten}+b_{1}\right)\\ x_{i}^{S} & =w_{2}z_{i}^{S}+b_{2}\\ p({\tilde{y}}_{i,j}^{S} & =1,j=1,2,\ldots ,c|x_{i}^{S})=\frac{\exp\left(x_{i,j}^{S}\right)}{\sum_{j=1}^{c}\exp\left(x_{i,j}^{S}\right)} \end{array}$$

where *c* is the number of labels. Here we only classify the labeled source feature representations $x_{i}^{S}$. The predicted vector can be written as ${\tilde{y}}_{i}^{S}=\left[\begin{array}{c} {\tilde{y}}_{i,0}^{S},{\tilde{y}}_{i,1}^{S},\ldots ,{\tilde{y}}_{i,c}^{S} \end{array}\right]$.

To measure the discrepancy between the source and target feature representations, a certain criterion $d(z^{S},z^{T})$ is chosen as a loss function. To achieve the purpose of domain adaptation, we minimize $d(z^{S},z^{T})$ and the classification error of source domain $\ell_{clf}\left(y^{S},{\tilde{y}}^{S}\right)$ simultaneously. Thus, the optimization objective of domain adaptation is expressed as [41]:

(15) $$\min_{\theta }\ell \left(y^{S},{\tilde{y}}^{S},z^{S},z^{T}\right)=\ell_{clf}\left(y^{S},{\tilde{y}}^{S}\right)+\lambda d\left(z^{S},z^{T}\right)$$

where λ is the regularization parameter, $\theta =\left\{\theta_{r_{j}}^{conv1},\theta^{conv2},\theta^{fc},\theta^{clf}\right\}$ represents the parameter set of FFCNN.

To optimize the network, we calculate the gradient of objective function with respect to network parameters and upgrade parameters according to the backpropagation (BP) algorithm and mini-batch stochastic gradient descent (SGD) algorithm [41]:

(16) $$\begin{array}{cc} \theta_{clf} & \leftarrow \theta_{clf}-\eta \frac{\partial \ell_{clf}}{\partial \theta_{clf}}\\ \theta_{fc} & \leftarrow \theta_{fc}-\eta \left(\frac{\partial \ell_{clf}}{\partial \theta_{fc}}+\lambda \frac{\partial d}{\partial \theta_{fc}}\right)\\ \theta_{fc}^{cov2} & \leftarrow \theta^{conv2}-\eta \left(\frac{\partial \ell_{clf}}{\partial \theta_{conv2}}+\lambda \frac{\partial d}{\partial \theta_{conv2}}\right)\\ \theta_{r_{j}}^{conv1} & \leftarrow \theta_{r_{j}}^{conv1}-\eta \left(\frac{\partial \ell_{clf}}{\partial \theta_{r_{j}}^{conv1}}+\lambda \frac{\partial d}{\partial \theta_{r_{j}}^{conv1}}\right) \end{array}$$

where η is the learning rate. The complete training process of FFCNN is shown in Algorithm 1.
**Algorithm 1** The algorithm of FFCNN back-propagation. **Input:** Labeled source domain samples ${\left\{(x_{i}^{S},y_{i})\right\}}_{i=1}^{m}$, unlabeled target domain samples ${\left\{(x_{i}^{t})\right\}}_{i=1}^{m}$, regularization parameter λ, learning rate η, dilate rate $\left\{r_{1},r_{2},r_{3}\right\}$. **Output** Network parameters $\left\{\theta_{r_{j}}^{conv1},\theta^{conv2},\theta^{fc},\theta^{clf}\right\}$ and predicted labels for target domain samples. **Begin:** Initialization for $\left\{\theta_{r_{j}}^{conv1},\theta^{conv2},\theta^{fc},\theta^{clf}\right\}$. **while** stopping criteria is not met **do**  **for** each source and target domain samples of mini-batch size $m^{\prime }$
**do**   Calculate output $x_{r_{j}}^{conv1}$ of each branch in dilate convolution layer according to    Equation (9).   Connect ${\left\{x_{r_{j}}^{conv1}\right\}}_{j=1}^{3}$, and calculate output of the second convolution layer according   to Equation (10).   Calculate features representations $z_{i}$ and output of softmax layer according to    Equation (11).   Calculate loss $\ell (y^{S},{\tilde{y}}^{S},z^{S},z^{T})$ according to Equation (12)   Upgrade $\left\{\theta_{r_{j}}^{conv1},\theta^{conv2},\theta^{fc},\theta^{clf}\right\}$ according to Equation (13).  **end for**
 **end while**

### 5.3. Diagnosis Procedure

**Figure 5:**
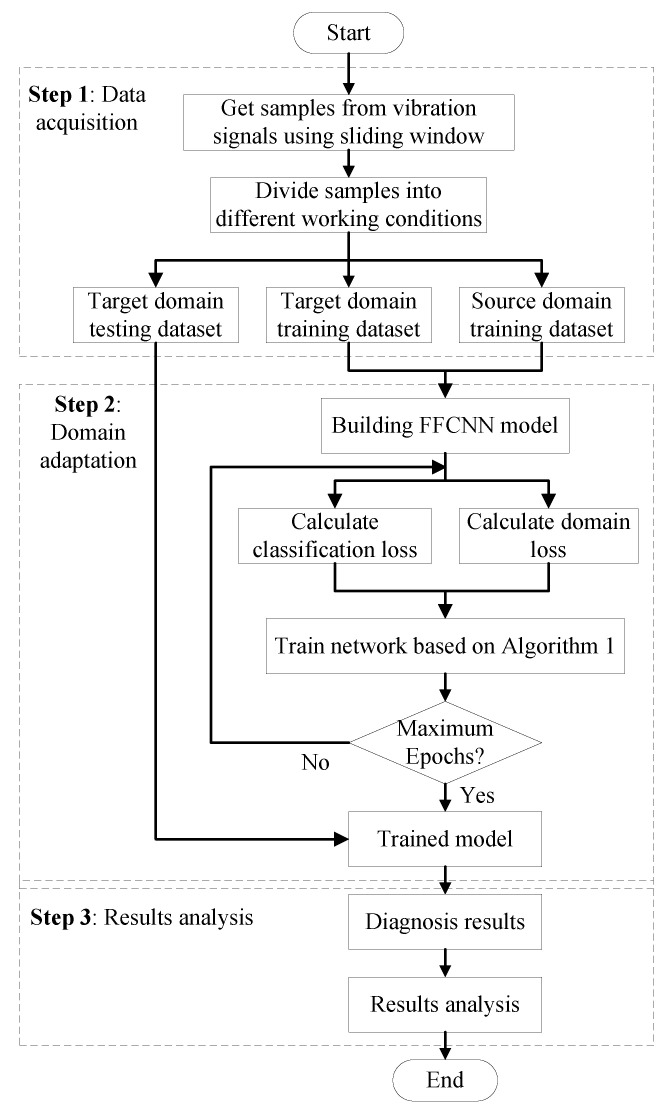
Flowchart of proposed FFCNN for fault diagnosis.

The flowchart of the proposed FFCNN for fault diagnosis is shown in Figure 5. It includes following two steps:

- Step 1: Data acquisition. The raw vibration signals are collected by sensors. Then the signals are sliced by a certain length of sliding window with a certain step size. When the samples are ready, they are divided into different working conditions according to the different operation settings. Among them, working condition *i* is the source domain, and working condition *j* is the target domain(i≠j). The samples in each working condition are further divided into training data and testing data. Section 6.1 will introduce the dataset used in this paper and the working conditions settings.
- Step 2: Domain adaptation. Based on the specific fault diagnosis problem and dataset information, the FFCNN configuration is chosen. The details of FFCNN used in this paper have been stated in Section 5.1. For training stage, FFCNN is trained by source training data and target training data based on Algorithm 1. For the testing stage, the target testing data are fed into trained FFCNN to get classification results.
- Step 3: Results analysis. The diagnosis results will be analyzed form three perspective: network architecture, feature representation and frequency domain.

## 6. Experiment

### 6.1. Introduction to Datasets

CWRU bearing dataset. This dataset is provided by Case Western Reserve University (CWRU) Bearing Data Center [42]. Four different bearing conditions are considered in this dataset: normal (N), ball fault (B), inner race (IR) fault, and outer race (OR) fault. Each fault was artificially damaged by electrical discharge machining. The vibration data are collected under different motor speeds at a sampling frequency of 12kHz or 48kHz. According to the sampling frequency and motor speed, the dataset is divided into six different working conditions, as shown in Table 2.

Paderborn dataset. This bearing dataset is provided by the Chair of Design and Drive Technology, Paderborn University [43]. There are three types of bearings: healthy bearings, artificially damaged bearings, and realistically damaged bearings. Artificially damaged bearings arise in inner race or outer race, and realistic damages occur in the form of pitting or plastic deformation. In this paper, we only focus on the diagnosis of the artificial damages. The vibration signals are collected under different load torque, radial force, and rotational speed at s sampling frequency of 64 kHz. According to these different working conditions, the dataset is divided into four different subsets, as showed in Table 3.

**Figure 6:**
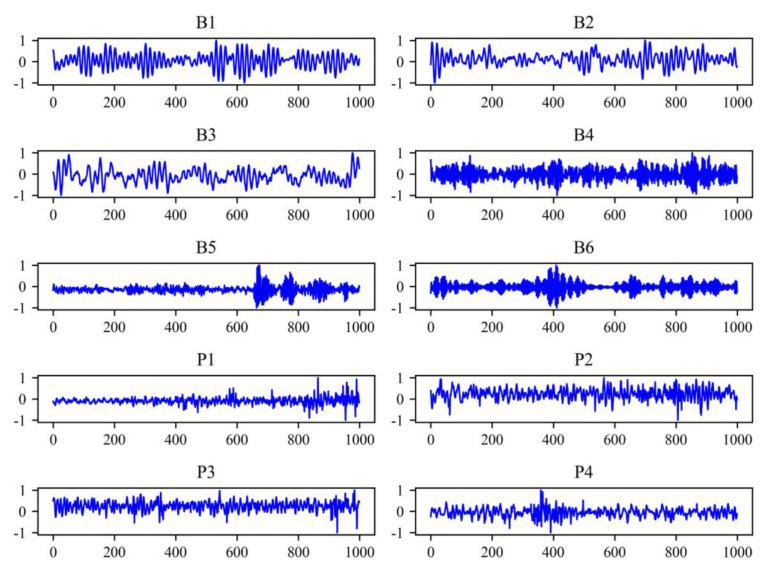
Example signals of CWRU and Paderborn dataset. B1 to B6 are the working conditions of CWRU dataset. P1 to P4 are the working conditions of Paderborn dataset.

Both above datasets are one-dimensional vibration signals, the example signals of CWRU and Paderborn dataset is shown in Figure 6. Because the length of the original signal is very long, the signals are sliced through a sliding window of length 1000, which means that each sample contains 1000 points. We use a sliding window with a sliding step size of 100 to get samples. For each fault type, we generate 1024 samples, and 20% of which are used as test sets.

### 6.2. Experiment Settings and Compared Methods

FFCNN is a method to improve the architecture of the domain adaptation network used in the feature representation based domain adaptation methods. These methods extract latent feature representations of the source domain and target domain, and reduce the discrepancy between them. Here we use three different discrepancy criterions: Maximum Mean Discrepancy (MMD), CORrelation ALignment (CORAL), and Central Moment Discrepancy (CMD).

- MMD: MMD criterion maps features to a Reproducing Kernel Hilbert Space (RKHS) to measure the discrepancy between source and target domain [44]. It is defined as:
  (17) $$d_{MMD}\left(z^{S},z^{T}\right)={∥\frac{1}{n_{S}}\sum_{i=1}^{n_{s}}\phi \left(z_{i}^{S}\right)-\frac{1}{n_{T}}\sum_{j=1}^{n_{T}}\phi \left(z_{i}^{T}\right)∥}_{H}$$
  where ϕ(·):Z→H is referred to as the feature space map.
- CORAL: CORAL criterion measures the discrepancy using the second-order statistics of source and target domain feature representations [45]. It is defined as:
  (18) $$\begin{array}{cc} \; & d_{CORa\;L}\left(z^{S},z^{T}\right)=\frac{1}{4d^{2}}{∥C_{S}-C_{T}∥}_{F}^{2}\\ \; & C_{S}=\frac{1}{n_{S}-1}\left(z^{S⊤}z^{S}-\frac{1}{n_{S}}{\left(1^{⊤}z^{S}\right)}^{⊤}\left(1^{⊤}z^{S}\right)\right)\\ \; & C_{T}=\frac{1}{n_{T}-1}\left(z^{T⊤}z^{T}-\frac{1}{n_{T}}{\left(1^{⊤}z^{T}\right)}^{⊤}\left(1^{⊤}z^{T}\right)\right) \end{array}$$
  where 1 is a vector with all elements equal to 1.
- CMD: CMD criterion matches the domains by explicitly minimizing differences of higher order central moments for each moment order [41]. It is defined as:
  (19) $$\begin{array}{c} d_{CMD}\left(z^{S},z^{T}\right)=\frac{1}{\left|b-a\right|}∥E\left(z^{S}\right)-E\left(z^{T}\right)∥\\ +\sum_{k=2}^{K}\frac{1}{{\left|b-1\right|}^{k}}{∥C_{k}\left(z^{S}\right)-C_{k}\left(z^{S}\right)∥}_{2} \end{array}$$
  where $E\left(z^{S}\right)=\frac{1}{n_{S}}\sum_{i=1}^{n_{S}}z_{i}^{S}$ is empirical expectation vector computed on features $z^{S}$, and $C_{k}\left(z^{S}\right)=E\left({\left(z^{S}-E\left(z^{S}\right)\right)}^{k}\right)$ is the vector of all $k^{th}$ order samples central moments of the coordinates of $z_{i}^{S}$.

For FFCNN, we use two dilate rate settings to evaluate the influence of dilate rate, one is r=1,2,3 named FFCNN-A, and another is r=1,3,5 named FFCNN-B. Moreover, we compared FFCNN with the ordinary CNN under the same computational complexity. In the first layer of FFCNN, each branch has a kernel with 8 channels and a size of 15, so three branches are equivalent to have a kernel with 24 channels and a size of 15. To keep the same computational complexity, the first layer of ordinary CNN also has a kernel with 24 channels and a size of 15, and the other layers are the same as the FFCNN. Besides, we also give the direct test results of the target domain data on the model trained by source domain dataset, called source-only. In these experiments, we set the number of epochs to be 50 and batch size to be 64. Adam optimization algorithm and CosineAnnealingLR with an initial learning rate of 0.001 are applied. Five-fold cross-validation is used for each task. The code is implemented by Tensorflow 2.0 and run on Tesla K80 GPU.

### 6.3. Experiment Results

**Figure 7:**
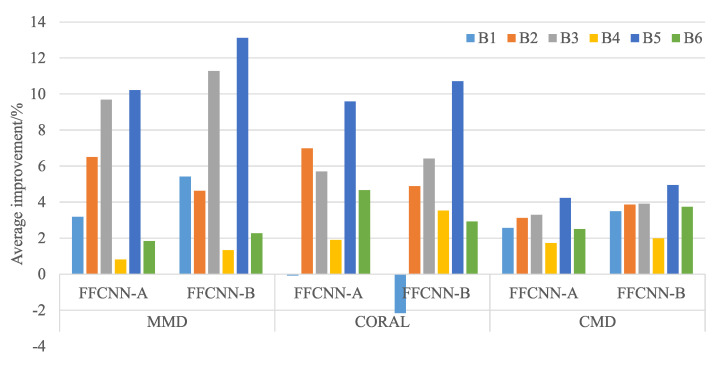
Average improvement of CWRU dataset.

**Figure 8:**
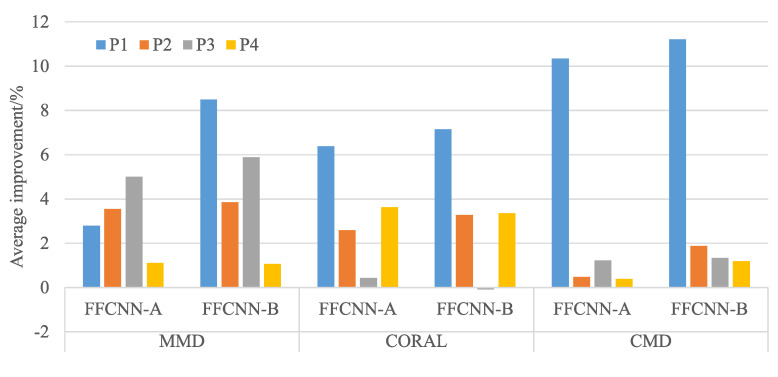
Average improvement of Paderborn dataset.

The diagnosis results using CWRU dataset are shown in Table 4, and results using Paderborn dataset are shown in Table 5. To show the improvement effect of FFCNN more clearly, we average the improved accuracy of FFCNN compared to normal CNN in each source domain. For example, source domain $B_{1}$ is transferred to five target domain $B_{j}\left(j=2,3,4,5,6\right)$, the improved accuracies of FFCNN compared with CNN are averaged. The results are shown in Figure 7 and Figure 8. We can see that the diagnostic accuracy of FFCNN in most tasks is significantly improved compared to CNN. Only the average effect of FFCNN-B using CORAL in CWRU dataset has not improved. Next, we will illustrate and analyze the results from three aspects in depth.

**Figure 9:**
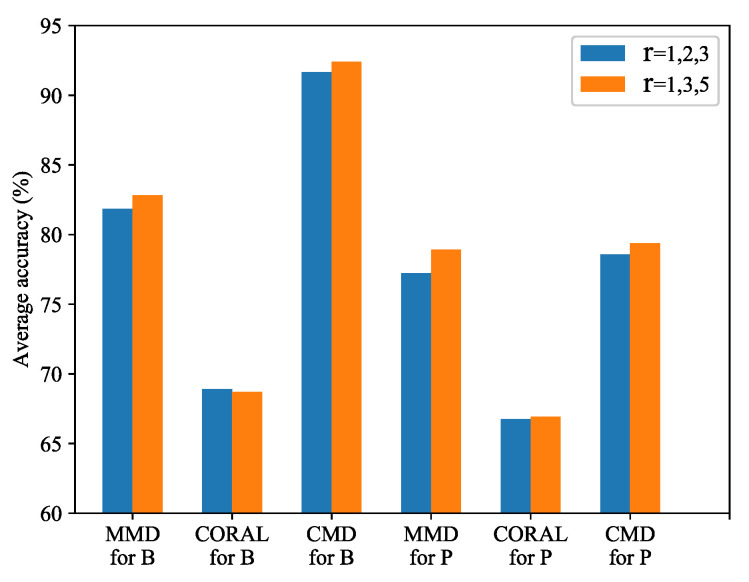
Average accuracy of FFCNN with different dilate rate on all tasks. B tasks are the tasks evaluated on CWRU dataset, and P tasks are the tasks evaluated on Paderborn dataset.

- The effectiveness of domain adaptation. These tables show that source-only, without domain adaptation, performs poorly. In comparison, domain adaptation methods greatly exceed source-only in most tasks. For example, in task B1→B4, the accuracy of source-only is 30.32%, but the accuracy of domain adaptation is 75.15% at the lowest and 100% at the highest. But domain adaptation fails in some cases. Such as task B2→B3, the accuracy of source-only is 72.27%, compared with 49.8% for CNN-MMD, 60.91% for FFCNN-A, and 55.15% for FFCNN-B. We suppose that these two methods did not extract the appropriate features to adapt the source domain and target domain. Overall, domain adaptation methods achieved the highest average accuracy, proving the strong generalization of domain adaptation.
- The effectiveness of FFCNN. FFCNN used different dilation rates to extract features at different scales, so that it may extract better features. Compared with ordinary CNN, FFCNN is more effective in most tasks. In some tasks, the effect of using FFCNN can be greatly improved. For example, in task B5→B1, FFCNN-B improved by 17.34% compared with CNN-MMD, 22.11% compared with CNN-CORAL, and 12.33% compared with CNN-CMD. But FFCNN may not be effective in some cases, such as FFCNN-A compared with CNN-MMD and FFCNN-B compared with CNN-CORAL in task B5→B3. For some tasks, a feature extracted at a fixed scale may be the most significant, but multi-scale convolution may weaken the influence of such a significant feature. Nevertheless, FFCNN performs well both in terms of the accuracy for most individual tasks and the average accuracy for all tasks.
- The influence of dilation rate. To clearly illustrate the effect of dilation rate, the average accuracy of FFCNN with different dilation rates on all tasks is shown in Figure 9. As directed from the figure, FFCNN with r=1,3,5 performs better than FFCNN with r=1,2,3, except CORAL for B tasks. According to Equation (8), the kernels of size H=15 with dilation rate r=1,2,3,4,5 are equivalent to the kernels of size $H_{dilated}=15,29,43,57,71$. It can be concluded that a large dilation rate has a larger receptive field, which can improve the effect of domain adaptation. Further analysis of dilation rate and dilated convolution will be discussed in the following sections.
- Dilated convolution v.s. common convolution. Dilated convolution expands the receptive field by expanding the convolution kernel. According to Equation (8), the receptive fields of different dilation rates and the receptive fields of specific size convolution kernels are equivalent. To show the advantage of dilated convolution, take task B5→B1 as an example, dilated convolution and common convolution are applied on CNN and FFCNN. The number of parameters and diagnosis accuracy of dilated convolution and common convolution are compared. The results are shown in Table 6. As we can see, the models using dilated convolution with different dilation rates do not increase the number of parameters. In general, their accuracy is higher than the models using common convolution kernels. This shows that both in terms of model size and diagnosis accuracy, dilated convolutions have advantages over common convolutions.

### 6.4. Analysis

#### 6.4.1. Analysis from the Perspective of Network Architecture

**Figure 10:**
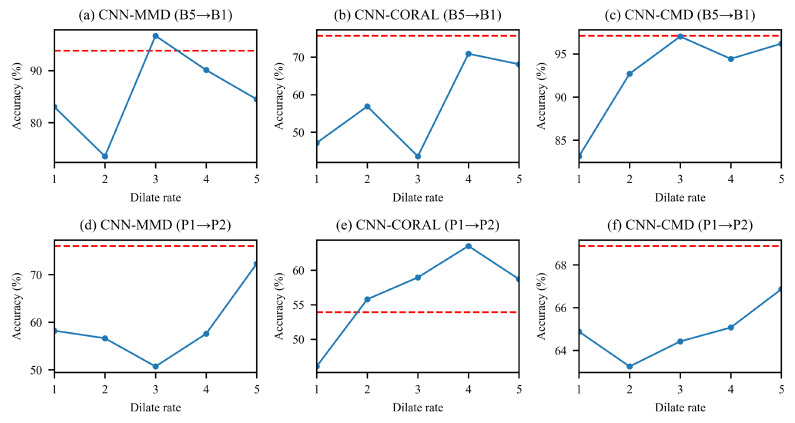
The accuracy under different dilation rates in the first layer of ordinary CNN.

FFCNN extracts features from multi scales using dilated convolution without increasing computational complexity, and different dilation rates represent different scales of the receptive field. To show the effect of frequency-domain fusing convolution, the performance of different single scale CNN is shown in Figure 10. Each point in the figure represents the diagnosis accuracy with a single scale on a given task. Here we select task B5→B1 and P1→P2 as examples to change the dilation rate of the first convolution layer based on of CNN-MMD, CNN-CORAL, and CNN-CMD. The dilation rates on the horizontal axis are r=1,2,3,4,5, respectively. The dotted red line indicates the highest accuracy of FFCNN for the task in Section 5.3. As we can see, increasing the dilation rate may increase accuracy and may also result in a decrease in accuracy. But in most cases, it will not exceed the accuracy of FFCNN. Furthermore, we cannot know exactly which scale under the current task will get higher accuracy. Therefore, single scale convolution cannot be adapted to extract features to obtain better and more stable performance. On the other hand, FFCNN can fuse multi-scale information to extract richer features and obtain excellent and stable results in most cases.

#### 6.4.2. Analysis from the Perspective of Feature Representation

**Figure 11:**
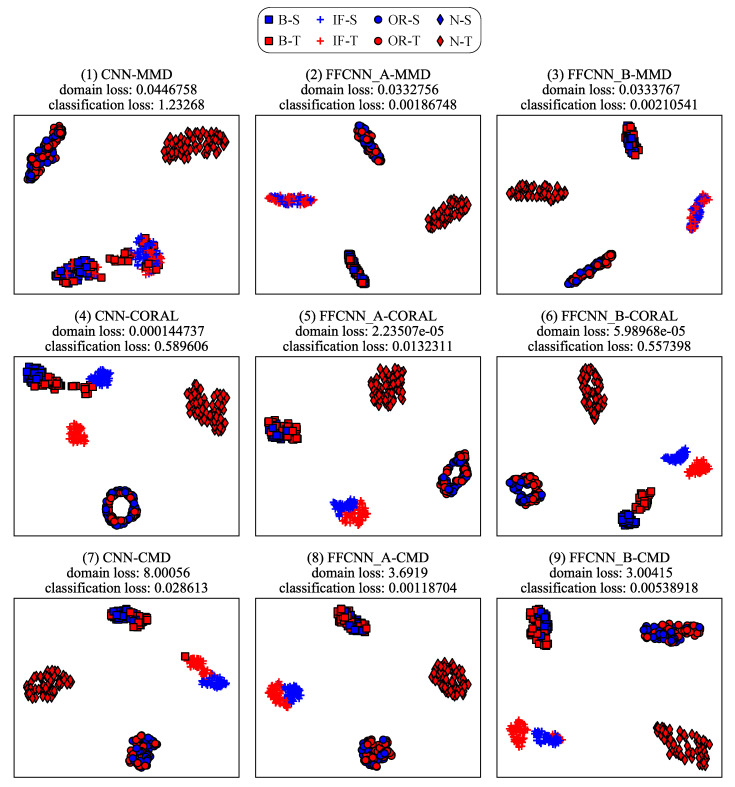
The visualization of learned features on CWRU dataset. The blue markers represent the source domain, the red markers represent the target domain. They are obtained from task B4→B5.

**Figure 12:**
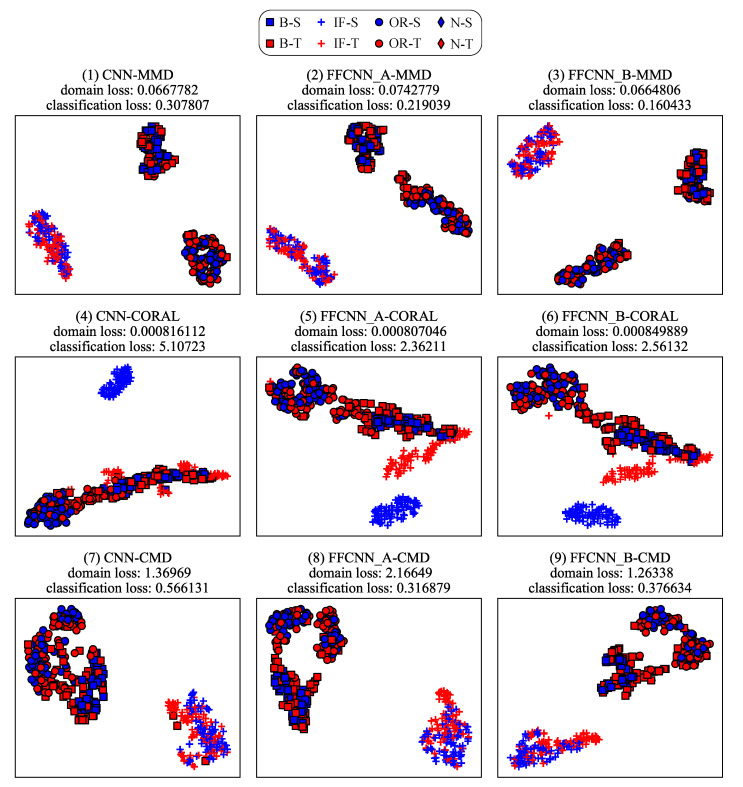
The visualization of learned features on Paderborn dataset. The blue markers represent the source domain, the red markers represent the target domain. They are obtained from task P3→P2.

Domain adaptation aims to align features of different domains. That is to say, domain adaptation will reduce the classification loss of source domain as well as the discrepancy between the source domain and target domain (called domain loss). So the features of different categories from the same domain can be dispersed as much as possible, and features of the same category from different domains can be gathered as much as possible. To illustrate the effectiveness of FFCNN from this perspective, we use task B4→B5 and P3→P2 as examples to visualize the features after the adaptation using t-SNE algorithm [46] in Figure 11 and Figure 12. For each subgraph, the domain loss and classification loss are also shown above. From the figures, we can see that the feature distributions of categories between the source domain and target domain are not aligned well without frequency-fusing method, such as ball fault and inner race fault in CNN-MMD of Figure 11. But under FFCNN framework, the improvement of distribution adaptation is noticeable. For example, in CNN-MMD of Figure 11, categories of source domain or target domain are separated, but didn’t align the feature distributions of the same category between source and target domain. On the contrary, FFCNN-A-MMD successfully aligns the feature distributions between domains, and the domain loss is $3.32756\times 10^{-2}$, which is better than $4.46758\times 10^{-2}$ of CNN-MMD. This improvement has raised the accuracy of CNN-MMD from 80.98% to 94.80%, and reduced the classification loss from 1.23268 to $1.86748\times 10^{-3}$. Similarly, the improvement of aligning effect will improve accuracy in other tasks.

#### 6.4.3. Analysis from the Perspective of Frequency Domain

**Figure 13:**
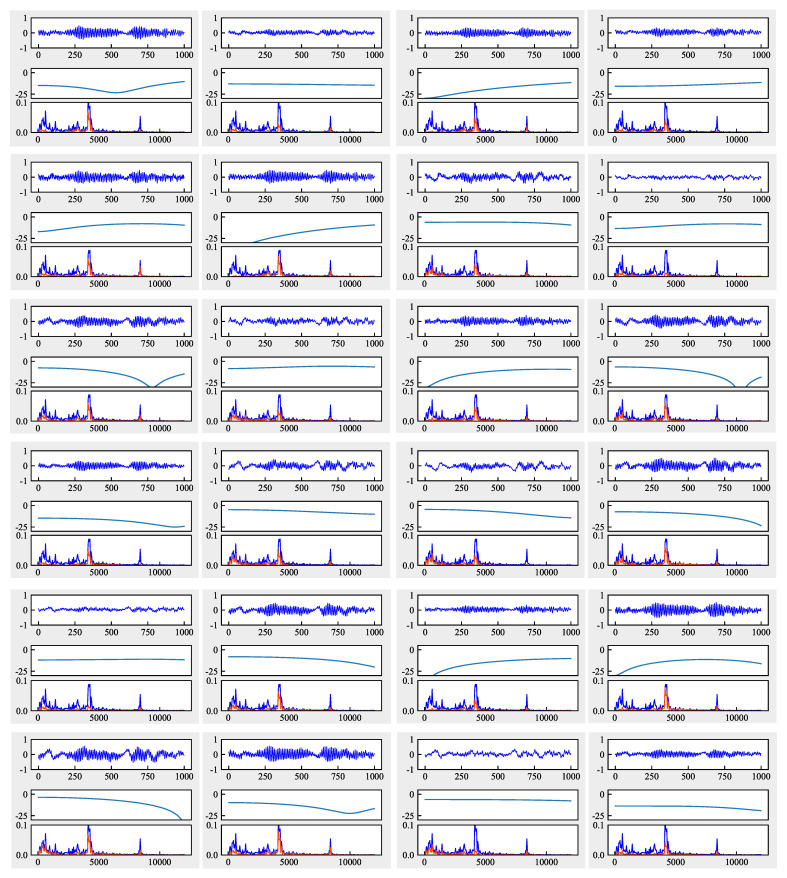
Amplitude-frequency characteristic curves of each filter in the first layer of CNN-maximum mean discrepancies (MMD) from task B5→B1.

**Figure 14:**
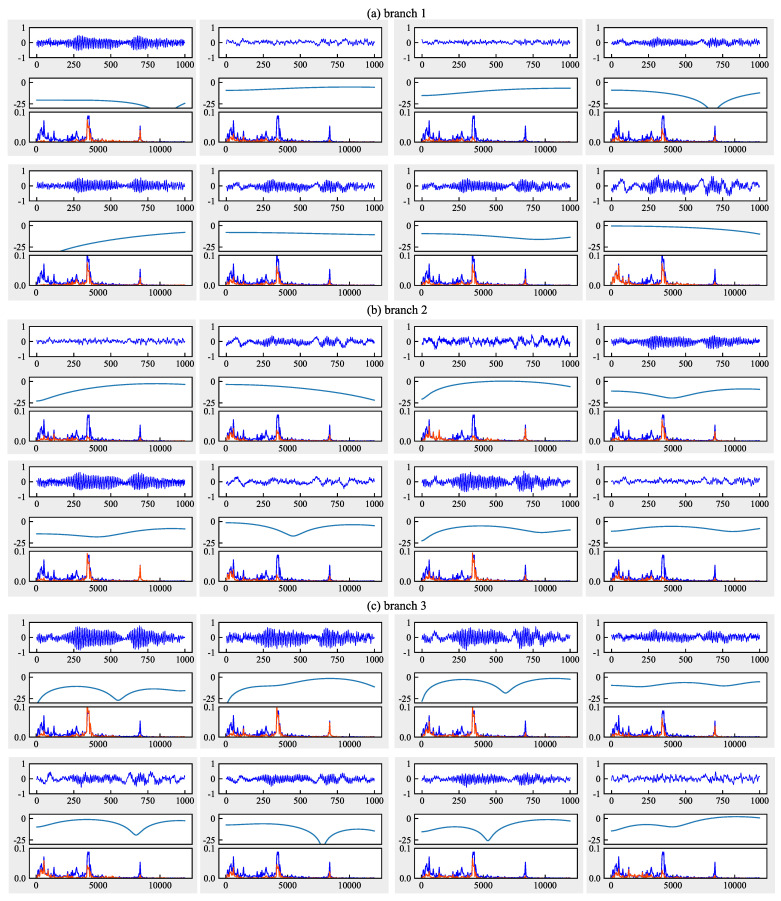
Amplitude-frequency characteristic curves of each filter in the first layer of FFCNN-A from task B5→B1. (**a**–**c**) represent the branches 1, 2, 3 with a dilation rate = 1, 2, 3, respectively.

**Figure 15:**
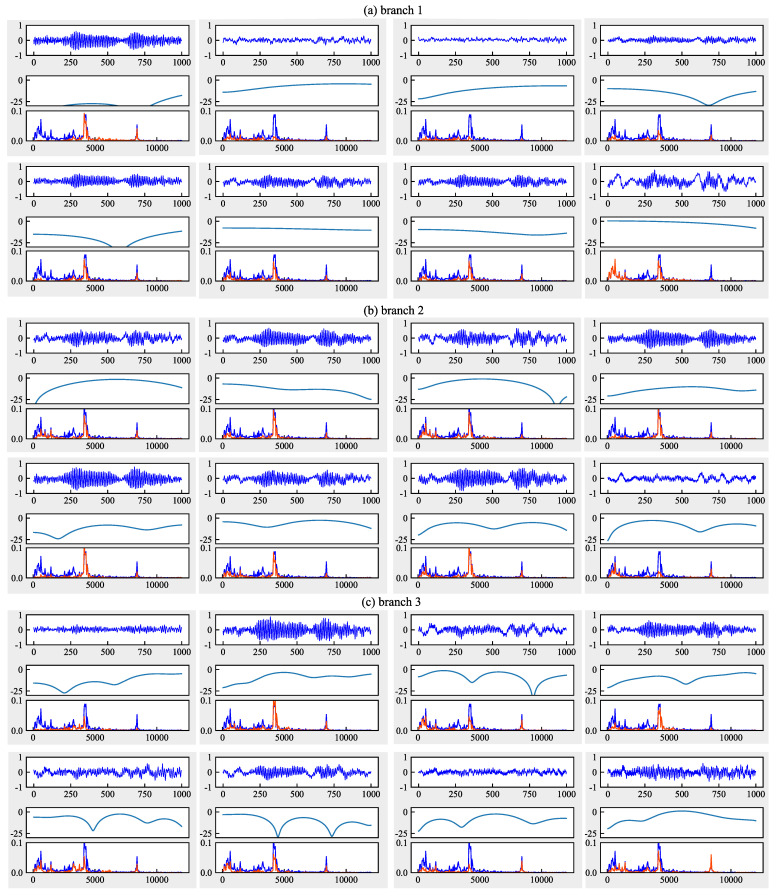
Amplitude-frequency characteristic curves of each filter in the first layer of FFCNN-B from task B5→B1. (**a**–**c**) represent the branches 1, 2, 3 with a dilation rate = 1, 3, 5, respectively.

Figure 13, Figure 14 and Figure 15 give the convolved signals, AFC curves, and FFT spectrogram of each filter in the first layer of CNN-MMD, FFCNN-A, and FFCNN-B from task B5→B1. Signals, AFC curve and FFT spectrogram form a sub-figure in a figure vertically. For the FFT spectrogram, the blue curve represents the FFT of input signal, and red represents the FFT of convolved signal. Combining FFT spectrogram, We can see that, compared with multi-scale convolution, the frequency band perceived by ordinary CNN is single. Signals filtered by different frequency bands will contain more significant useful information, and frequency bands that do not contribute to fault classification will be suppressed. During the training process, the network will learn which frequency bands are useful and which are not according to the loss function changes.

## 7. Discussion

This paper has proved the effectiveness of FFCNN with a large number of experiments and explained it from multiple perspectives. For the application of FFCNN, we have the following suggestions:

- FFCNN is a unified domain adaptation architecture for fault diagnosis, it can also be applied to other CNN structures, domain adaptation methods or datasets.
- Which dilation rates are used to construct a FFCNN need to be determined according to the specific task, not necessarily r=1,2,3 or r=1,3,5. And the number of combined scales can also change.
- AFC curve can be considered as a general CNN analysis method. It provides a new perspective for describing the characteristics of the convolution kernel.
- Multi-scale convolution kernels are generally applied in the first layer, and using multi-scale convolution in the middle layers has not been studied to prove its effectiveness.

While FFCNN is effectively applied in domain adaptation for fault diagnosis, we still face the following challenges regarding transfer learning and fault diagnosis:

- While FFCNN can improve the effect of domain adaptation, if the source domain and target domain are too different, FFCNN will also fail. How to further enhance the effect of domain adaptation still needs to be further studied [47].
- We explained the FFCNN from the perspective of frequency domain. How to improve the interpretability of deep learning methods for fault diagnosis is a more challenging task [13].

## 8. Conclusions

In this paper, a unified CNN architecture for domain adaptation named FFCNN using dilated convolutions with different scale is proposed. Experiments on two bearing datasets have proved the significant effect of FFCNN. Based on the results and analysis, three main significances of this paper can be concluded. First, the proposed FFCNN is driven from the perspective of frequency-domain characteristic. This inspires researchers to combine frequency-domain analysis with neural networks. Second, the frequency domain characteristic is described by the AFC curve, providing a new means to understand CNN. Third, results on different domain loss functions show that FFCNN is suitable for various domain adaptation losses. Thus, FFCNN provides an example for unified domain adaptation network design. While the proposed FFCNN has certain interpretability, it still does not fully explain the working principle of CNN. Further understanding of CNN to improve the effectiveness of fault diagnosis will be future work.

## Figures and Tables

**Table 1:** Details of proposed Frequency-domain Fusing Convolutional Neural Network (FFCNN) architecture.

Layer	Hyperparameters
CONV ($r_{1}$)	$r_{1}=1$; channels: 8, kernel size: 15; stride: 1; activation: ReLu; padding: same
CONV ($r_{2}$)	$r_{2}=2$ (or 3); channels: 8, kernel size: 15; stride: 1; activation: ReLu; padding: same
CONV ($r_{3}$)	$r_{3}=3$ (or 5); channels: 8, kernel size: 15; stride: 1; activation: ReLu; padding: same
POOL1	Average Pooling, stride: 2
CONV	channels: 32, kernel size:15; stride: 1; activation: ReLu; padding: same
POOL2	Average Pooling, stride: 2
Features layer	Node number: 256, activation: ReLu
Softmax layer	Node number: number of faults types, activation: softmax

**Table 2:** Working conditions settings of Case Western Reserve University (CWRU) dataset.

Sampling Frequency	Sensor Position	Speed (rpm)	Name of Setting
48 kHz	Driven end	1796	B1
48 kHz	Driven end	1772	B2
48 kHz	Driven end	1725	B3
12 kHz	Driven end	1796	B4
12 kHz	Driven end	1725	B5
12 kHz	Driven end	1750	B6

**Table 3:** Working conditions settings of Paderborn dataset.

Rotating Speed (rpm)	Load Torque (Nm)	Radial Force (N)	Fault Type	Name of Setting
900	0.7	1000	Health, inner fault, outer fault	P1
1500	0.1	1000		P2
1500	0.7	400		P3
1500	0.7	1000		P4

**Table 4:** Diagnosis accuracy (%) on different working conditions compared with different methods using CWRU dataset. The values in bold indicate that FFCNN has a higher accuracy rate than CNN.

Tasks	Source Only	CNN-MMD	FFCNN-A	FFCNN-B	CNN-CORAL	FFCNN-A	FFCNN-B	CNN-CMD	FFCNN-A	FFCNN-B
B1→B2	75.10	81.13	**89.65**	**90.28**	75.20	75.17	**75.44**	78.49	**81.91**	**83.64**
B1→B3	78.69	79.27	**81.96**	**84.15**	79.32	**81.66**	**83.77**	82.86	**87.06**	**90.09**
B1→B4	30.32	98.32	**100.00**	98.12	75.15	74.66	61.69	97.83	**99.44**	**99.78**
B1→B5	31.13	67.48	**70.48**	**80.76**	66.90	65.19	**71.26**	92.90	**96.92**	**96.19**
B1→B6	48.73	100.00	**100.00**	**99.98**	76.86	76.39	70.46	99.46	99.00	99.29
B2→B1	88.13	90.21	**98.66**	**99.63**	89.82	**93.46**	**95.97**	90.80	**94.41**	**96.12**
B2→B3	72.27	49.80	**60.91**	**55.15**	73.54	**76.42**	**74.27**	73.49	**74.93**	**75.17**
B2→B4	50.00	97.05	**97.05**	96.12	57.03	**68.43**	**66.58**	97.51	**98.66**	**98.68**
B2→B5	50.00	54.90	**65.31**	**60.77**	50.54	**53.37**	49.71	89.62	**98.17**	**97.12**
B2→B6	40.40	55.91	**58.42**	**59.30**	35.33	**49.52**	**44.14**	95.80	**96.63**	**99.44**
B3→B1	60.76	99.95	**100.00**	**100.00**	76.59	**92.28**	**96.66**	99.56	**99.88**	**99.98**
B3→B2	54.30	66.35	**67.01**	**74.51**	61.13	**69.80**	**72.63**	75.85	74.85	73.00
B3→B4	50.00	75.02	**86.62**	**85.86**	50.00	**50.00**	**50.00**	89.19	**95.85**	**98.15**
B3→B5	51.25	59.15	**96.02**	**97.37**	51.95	51.42	**52.00**	86.55	**92.82**	**95.68**
B3→B6	49.54	99.95	99.22	99.10	49.58	**54.24**	**50.05**	95.14	**99.34**	**99.05**
B4→B1	25.71	100.00	**100.00**	99.19	86.33	84.15	**86.52**	98.90	**99.95**	**99.90**
B4→B2	33.45	75.63	75.22	74.98	73.02	**74.85**	**74.05**	76.49	**76.66**	76.29
B4→B3	38.53	59.23	**59.30**	**62.28**	47.00	**56.47**	**65.11**	70.26	**77.54**	**79.66**
B4→B5	58.89	80.98	**94.80**	**95.48**	85.25	**90.23**	**93.66**	99.39	**99.56**	99.10
B4→B6	78.05	100.00	90.57	90.59	94.55	89.97	84.45	100.00	**100.00**	**100.00**
B5→B1	26.41	76.41	**86.23**	**93.75**	53.57	**61.45**	**75.68**	84.84	**92.58**	**97.17**
B5→B2	25.46	46.09	**54.34**	**52.22**	38.91	**48.68**	**47.04**	72.70	**79.10**	**75.12**
B5→B3	35.65	76.44	71.07	**79.57**	66.95	**67.14**	56.86	70.51	**77.51**	**80.47**
B5→B4	50.07	51.88	**70.55**	**71.32**	50.00	**69.19**	**73.02**	99.95	**100.00**	**100.00**
B5→B6	50.07	52.39	**72.10**	**71.97**	76.66	**87.60**	**87.01**	100.00	**100.00**	**100.00**
B6→B1	25.00	95.53	**95.56**	**100.00**	46.24	42.62	**52.88**	98.32	**99.12**	**99.93**
B6→B2	25.00	59.50	59.42	58.88	36.45	**40.87**	**48.34**	70.38	**77.66**	**76.73**
B6→B3	35.84	70.07	**77.63**	**82.32**	63.38	61.91	51.93	77.03	**80.86**	**87.04**
B6→B4	51.56	100.00	**100.00**	**100.00**	75.00	**75.00**	74.98	100.00	**100.00**	**100.00**
B6→B5	54.00	76.00	**77.66**	71.26	67.53	**85.18**	**75.12**	99.02	**99.63**	**99.73**
AVG	48.14	76.49	**81.86**	**82.83**	64.33	**68.91**	**68.71**	88.76	**91.67**	**92.42**

**Table 5:** Diagnosis accuracy (%) on different working conditions compared with different methods using Paderborn dataset. The values in bold indicate that FFCNN has a higher accuracy rate than CNN.

Tasks	Source Only	CNN-MMD	FFCNN-A	FFCNN-B	CNN-CORAL	FFCNN-A	FFCNN-B	CNN-CMD	FFCNN-A	FFCNN-B
P1→P2	42.71	56.09	**69.47**	**76.33**	46.65	**51.95**	**53.48**	62.66	**68.94**	**65.79**
P1→P3	50.62	18.07	**18.30**	**20.61**	57.72	**65.04**	**64.94**	42.28	**59.80**	**64.42**
P1→P4	41.57	51.31	46.07	**54.00**	46.39	**52.90**	**53.78**	54.75	**61.98**	**63.15**
P2→P1	48.92	76.78	**88.57**	**87.37**	52.63	**61.33**	**62.24**	72.79	**74.64**	**76.30**
P2→P3	87.05	94.47	**95.15**	**94.89**	90.46	**92.35**	**92.48**	93.13	**93.78**	**93.16**
P2→P4	88.28	91.96	90.14	**92.51**	88.64	85.81	86.85	88.64	87.60	**90.72**
P3→P1	39.81	65.09	**80.25**	**81.24**	39.06	**40.23**	**40.53**	74.09	**74.97**	**75.91**
P3→P2	57.62	92.12	**92.90**	**93.88**	62.77	**65.10**	**65.40**	87.21	**89.78**	**90.40**
P3→P4	51.63	86.20	85.25	85.94	51.40	49.19	47.04	79.85	**80.08**	**78.87**
P4→P1	47.07	70.60	**74.58**	**72.69**	50.13	**59.11**	**56.93**	68.52	**70.28**	**71.48**
P4→P2	94.73	95.74	**96.09**	**96.71**	95.02	93.46	94.60	94.73	93.98	94.30
P4→P3	60.32	90.82	89.81	**90.95**	81.05	**84.51**	**84.73**	87.04	**87.21**	**88.09**
AVG	59.19	74.10	**77.22**	**78.93**	63.49	**66.75**	**66.92**	75.47	**78.59**	**79.38**

**Table 6:** Results of dilated convolution and common convolution.

CNN ${}^{1}$					
Dilated kernels ${}^{1}$			Common kernels		
Diration rate	Params ${}^{2}$	Acc	Kernel size	Params ${}^{2}$	Acc
1	11936	83.03	15	11936	83.03
2	11936	73.55	29	12272	**89.3**
3	11936	**96.65**	43	12608	64.85
4	11936	**90.09**	57	12944	68.58
5	11936	**84.48**	71	13280	83.87
**FFCNN**					
1, 2, 3	11936	86.23	15, 29, 43	12272	**88.06**
1, 3, 5	11936	**93.75**	15, 43, 71	12608	87.11

^1^ For fair comparison, dilated convolution and common convolution kernels of varying size only act on the first layer in CNN. ^2^ Only count the number of parameters in the convolutional layers.

## Abbreviations

T	Classification task
D	a specific domain
$D^{S},D^{T}$	Source domain and target domain
X	Input sample space
Y	Input label space
$X^{S},X^{T}$	Input source sample space and target sample space
$Y^{S},Y^{T}$	Input label sample space and target label space
X,Y	Dataset and labels
x,y	a sample and a label in dataset
*Z*	Learned features representation
g(·)	Feature extractor of deep learning model
h(·)	Classifier of deep learning model
$\ell_{clf},d\left(\cdot \right)$	classification loss and domain loss
G(·)	a convolution operation
$A(f_{i})$	Amplitude frequency characteristic of G(·) under frequency $f_{i}$
